# Long-term consumption of liquid dairy products predicts lower fracture risk in aging women: a 25-year follow-up

**DOI:** 10.1007/s00394-025-03709-7

**Published:** 2025-06-09

**Authors:** Fatemeh Ramezan Alaghehband, Arja T. Lyytinen, Masoud Isanejad, Juho Kopra, Heikki Kröger, Toni Rikkonen

**Affiliations:** 1 Institute of Public Health and Clinical Nutrition, University of Eastern Finland, Kuopio, 70211 Finland; 2 Institute of Life Course and Medical Science, Department of Musculoskeletal and Ageing Science, University of Liverpool, Liverpool, 6 8TX England; 3 Kuopio Musculoskeletal Research Unit, University of Eastern Finland, Kuopio, 70211 Finland; 4 Department of Orthopaedics and Traumatology, Kuopio University Hospital, Kuopio, 70210 Finland

**Keywords:** Dairy products, Milk, Cheese, Fracture, Osteoporotic fracture, Hip fracture

## Abstract

**Purpose:**

The study aimed to investigate whether high dairy products consumption is associated with reduced fracture risk in aging women, contributing to understanding this health issue.

**Method:**

Data was obtained from the Kuopio Osteoporosis Risk Factor and Prevention (OSTPRE) study, a large cohort of 14,220 older Finnish women (mean baseline age 52.3 years) with 25 years of follow-up. Participants completed questionnaires every five years, providing information on health status, lifestyle habits, dairy products consumption (milk, sour milk, yogurt, cheese), and fracture history. Cox proportional hazard models with time-dependent covariates were used to estimate hazard ratios for any fracture, hip fracture, or osteoporotic fracture based on dairy products consumption. The models were adjusted for time-updated BMI (kg/m²), alcohol use (portions/day), physical activity (hours/month), age (years), use of calcium and/or vitamin D supplements (yes/no), and use of bone-affecting medications (yes/no).

**Results:**

Higher liquid dairy products consumption (milk, sour milk, yogurt) was associated with decreased risk for any fracture (β = 0.98, SE = 0.01, *P* = 0.008) and osteoporotic fracture (β = 0.97, SE = 0.01, *P* = 0.011). In contrast, cheese consumption was not associated with the overall risk of any fracture or osteoporotic fracture. In a separate analysis, higher cheese consumption was linked to a reduced hip fracture risk (β = 0.91, SE = 0.05, *P* = 0.043) while no association was found between liquid dairy products consumption and hip fracture risk.

**Conclusion:**

A long-term consumption of liquid dairy products may lower fracture risk. Encouraging the consumption of these products through public health initiatives may help reduce fractures and ease the economic burden on the healthcare system.

## Introduction

Globally, fracture is a major public health issue causing pain, disabilities, loss of independence, diminished quality of life and premature mortality among older adults [1–3]. Over the past 3 decades, the fracture incidence has increased by 33.4% worldwide, mainly due to the aging of the population [4, 5]. Fractures in older adults are the most common clinical manifestation of osteoporosis [6]. Worldwide, 9 million osteoporotic fractures happen annually which equates to one osteoporotic fracture every 3 s [7]. In Finland, the incidence of osteoporotic fractures is projected to rise by 33% between 2019 and 2034 [8]. Therefore, considerable preventive efforts have been made to prevent fractures by targeting underlying conditions that may predispose older adults to fracture. Among these underlying conditions, the consumption of dairy products has been extensively studied [9–22].

Dairy products contain calcium, protein and other bioactive nutrients that benefit bone health [9, 10]. however, studies investigating the link between dairy product consumption and fracture reported mixed findings. A recent systematic review and meta-analysis study concluded that higher consumption of milk cannot predict risk of any fracture [11]. Similarly, no association between milk and dairy product consumption and risk of hip fracture were reported by two other reviews published in 2020 [12, 13]. Conversely, some studies suggest that greater dairy product consumption may reduce fracture risk. For instance, a cohort study with median follow-up of 15.2 years reported that higher consumption of milk was associated with lower risk of osteoporotic fracture [14]. A reduced risk of osteoporotic fracture with higher total dairy and milk consumption were also claimed by another cohort study that followed 103,003 women for 24 years [15]. Adding to this ambiguity, a large Swedish cohort study with mean follow-up of 20.1 year reported that daily consumption of three or more glasses of milk was associated with an increased risk of any fracture [16]. Additionally, Michaelsson et al. found that women who consume 3 servings of milk per day had 60% higher risk of hip fracture compared with those who consumed lower than 1 serving of milk per day [16].

A reason for the inconsistencies in the literature could be the lack of sufficient data from long-term cohort studies that contained updated data on dairy product consumption and validated fractures. To address this gap, the present study aims to explore the association between the consumption of liquid dairy products and cheese, and risk of any fracture, osteoporotic fracture, and hip fracture among aging women over 25 years. We hypothesize that the higher consumption of liquid dairy products and cheese is associated with a reduced fracture risk.

## Methods

### Study design

This study was conducted by using data from the ongoing Kuopio Osteoporosis Risk Factor and Prevention (OSTPRE) study [23]. The OSTPRE is an ongoing population-based prospective cohort study. It was launched in 1989 to assess the risk factors for osteoporosis and fractures in women [24]. The participants of OSTPRE were all women born between 1932 and 1941 and living in the Kuopio region, Eastern Finland (*n* = 14220). Subjects received postal questionnaires about their health status, lifestyle habits, and fracture history in five-year intervals between 1989 (baseline) and 2019. The questionnaire response rate ranged between 80% and 93% [25].

### Assessment of dairy products

The assessment was based on the participant’s consumption amount of liquid dairy products such as milk, sour milk and yogurt (dl/day); and cheese (slice/day) in a self-reported questionnaire.

### Assessment of fracture

Fracture data were collected through questions regarding time of fracture, fracture site, place of treatment, and previous fracture history. All fracture reports were validated from medical records. High-energy fractures including traffic accidents, falls above 1 m height and pathologic fractures were excluded from the analysis. The proximal humerus, vertebrae, distal radius, and hip fractures were classified as osteoporotic fractures.

### Assessment of covariates

The body mass index (BMI) was based on the self-reported height and weight. Calcium and vitamin D supplement use was assessed across six questionnaires using slightly different formats. In some questionnaires, participants were asked directly about their use of calcium and vitamin D as medicinal or natural products, while in others, use was recorded by selecting from a list of doctor-prescribed medications. Based on these data, binary variables were created to indicate use of calcium and/or vitamin D supplements over time. Although the amount of alcohol consumption was not included in the baseline questionnaire, it was recorded in all follow-up questionnaires. The assessment of physical activity (PA) has some variation in follow-up questionnaires. On the baseline questionnaire, the number of hours spent on regular PA per week was asked. On the 5-year and 10-year follow-up, the question included the number of hours spent on different types of PA during winter and summer seasons. On the 15-year, 20-year and 25-year follow-up questionnaires, the frequency and number of hours spent in PA per week, were questioned. Based on these, we calculated corresponding variables for the number of hours spent on PA per month. Bone-affecting medication use was assessed across six questionnaires. In three questionnaires, participants were provided with a list of specific medications commonly affect bone health (for example, Fosamax, Opinate, Bonefos, Didronate and Evista), which they were instructed to review and indicate any they had used. In one questionnaire, a direct yes/no question was used. In the two other questionnaires, participants reported all prescribed medications in free text, which were later reviewed and categorized as bone-affecting medications when applicable. Based on these data, binary variables were created to indicate reported use of bone-affecting medications over time.

### Statistical analysis

Statistical analyses were performed using SPSS version 27 and R version 4.2.2. A threshold probability of 0.05 was used to determine statistical significance. We calculated the time at risk until the first fracture, death, last reply, or end of the follow-up, whichever occurred first. Kaplan Meier survival analysis was used to illustrate the absolute risk of fractures between categories of dairy products consumption at baseline. The Cox proportional hazard model with time dependent covariates were used to calculate the hazard ratios with their respective 95% confidence intervals for any fracture, hip fracture and osteoporotic fracture. Liquid dairy product consumption was categorized into three groups: no intake (0 dl/day), moderate intake (≤ median intake of 4 dl/day), and high intake (> median intake of 4 dl/day), with no intake as the reference category. Cheese consumption was categorized into no intake (0 slices/day), moderate intake (≤ median intake of 3 slices/day), and high intake (> median intake of 3 slices/day), with no intake as the reference category. All analyses were conducted in both unadjusted and adjusted models. Several potential confounders were examined in bivariate Cox regression analysis to determine an association between dairy consumption and fractures. The final confounders were time-updated BMI (kg/m2), alcohol use (continuous), amount of PA (hours/month), age (years), use of calcium and/or vitamin D supplements (yes, no), and use of bone-affecting medications (yes, no). All covariates were entered simultaneously in the models.

## Results

The cohort had an average follow-up period of 17.63 (8.23) years. The total follow-up time was 245,005 person-years, during which 4,358 women experienced any type of fracture, 2,326 had osteoporotic fractures, and 427 had hip fractures. The remaining fractures (approximately 40%) not classified as osteoporotic fracture included those of the clavicle, ribs, sternum, pelvis (excluding hip), sacrum, coccyx, patella, tibia, fibula, foot, hand, facial bones, and skull. The mean baseline age of the participants was 52.30 (2.91) years. Additionally, the all-cause mortality rate over the follow-up period was 22.69%. Table 1 summarizes the characteristics of study participants according to their baseline consumption of liquid dairy products and cheese. Of the total population, 6.5% did not consume liquid dairy products, 45.7% consumed moderate amounts of liquid dairy products (≤ median intake of 4 dl/day), and 47.8% consumed high amounts of liquid dairy products (> median intake of 4 dl/day) at baseline (Table 1). In terms of cheese consumption, 9.4% of the population did not consume cheese, 46.2% consumed moderate amounts (≤ median intake of 3 slices/day), and 44.4% consumed high amounts (> median intake of 3 slices/day) at baseline. Women with higher consumption of liquid dairy products had higher BMI. In contrast, those with higher cheese consumption had lower BMI. The use of vitamin D and/or calcium supplements among women who consumed high amounts of liquid dairy products was significantly 8.4% lower compared to those who did not consume liquid dairy products. Cheese consumption is higher among those who consume less liquid dairy products. There was no significant difference in other baseline characteristics between categories of liquid dairy products and cheese consumption.

### Any and osteoporotic fractures

**Fig. 1:**
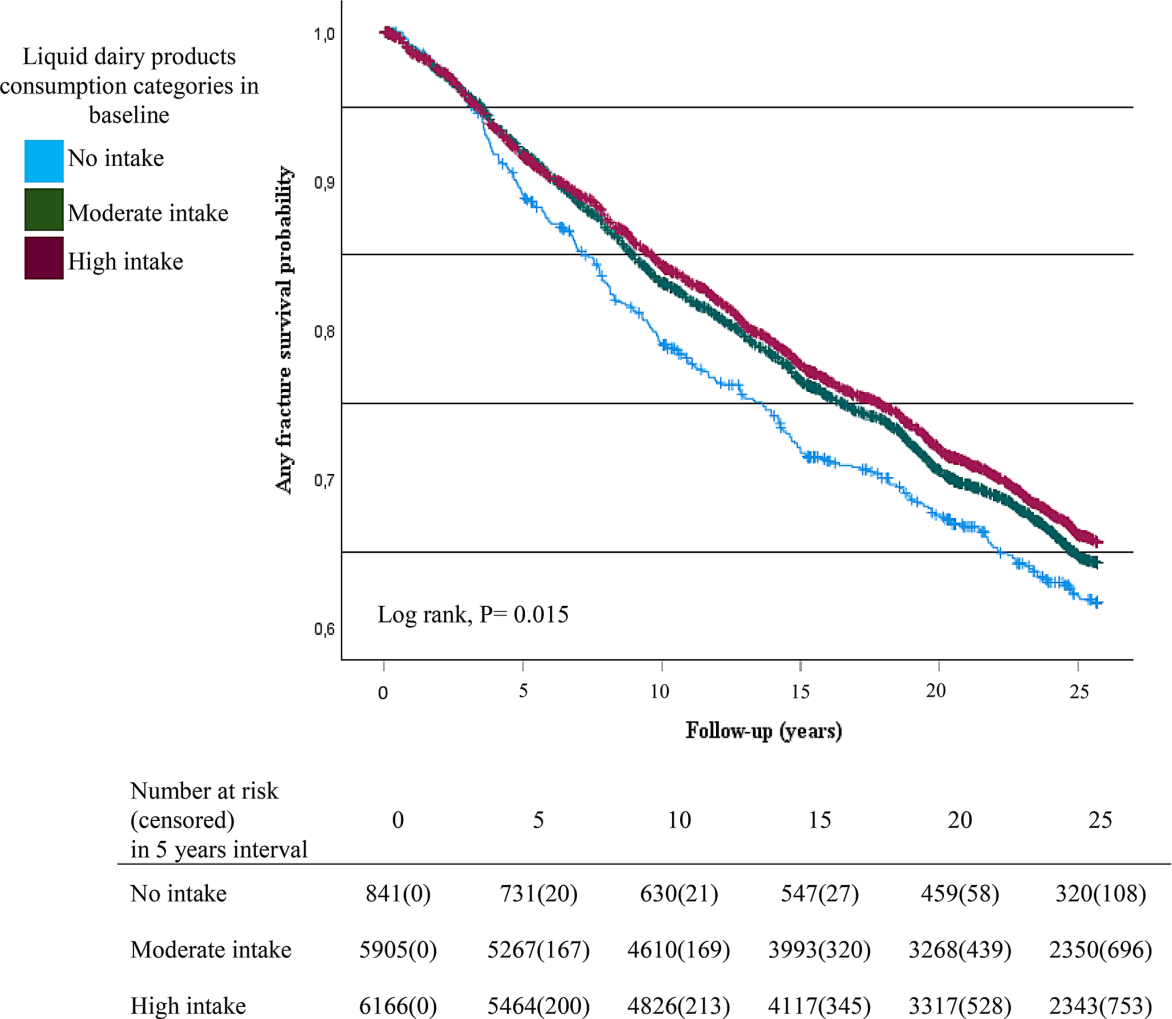
Kaplan Meier survival plot for any fracture in categories of liquid dairy products consumption

**Fig. 2:**
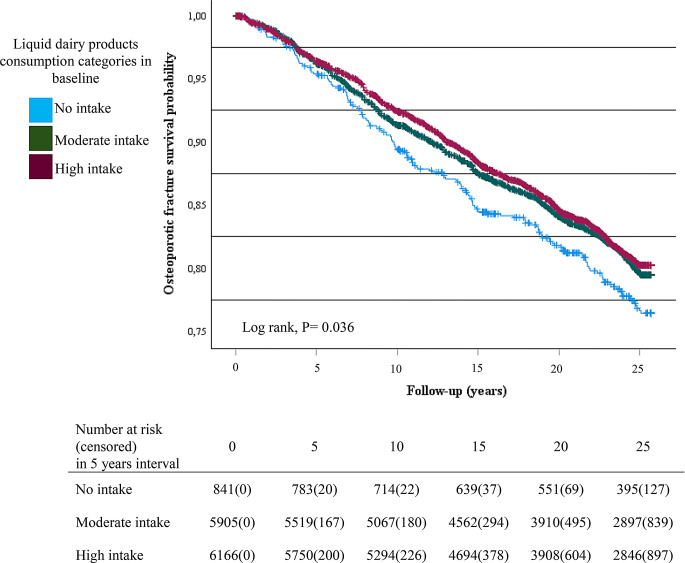
Kaplan Meier survival plot for osteoporotic fracture in categories of liquid dairy products consumption

According to the survival curves, women who did not consume liquid dairy products had a higher risk of any fracture and osteoporotic fracture compared to moderate or high consumers at baseline (Figs. 1 and 2). The hazard ratio for any fracture was 0.77 (95%CI = 0.66–0.94, P value = 0.002) in moderate consumers and 0.74 (95%CI = 0.62–0.87, P value < 0.001) in high consumers of liquid dairy products compared with no-consumers, when the analysis was done based on time-dependent covariates (Table 2). Similarly, the hazard ratio for osteoporotic fracture was 0.69 (95%CI = 0.56–0.86, P value = 0.001) in moderate consumers and 0.64 (95%CI = 0.51–0.80, P value < 0.001) in high consumers of liquid dairy products compared with no-consumers of liquid dairy products, when the analysis was done based on time-dependent covariates (Table 3). Furthermore, when the consumption of liquid dairy products was considered as a continuous variable, the higher consumption of liquid dairy products predicted a lower rate of any fracture (β = 0.98, 95%CI = 0.96–0.99, P value = 0.008) and osteoporotic fracture (β = 0.97, 95%CI = 0.95–0.99, P value = 0.011) in adjusted model with time-dependent covariates. Cheese consumption, however, did not predict the risk of any fracture or osteoporotic fracture (Tables 2 and 3).

### Hip fracture

**Fig. 3:**
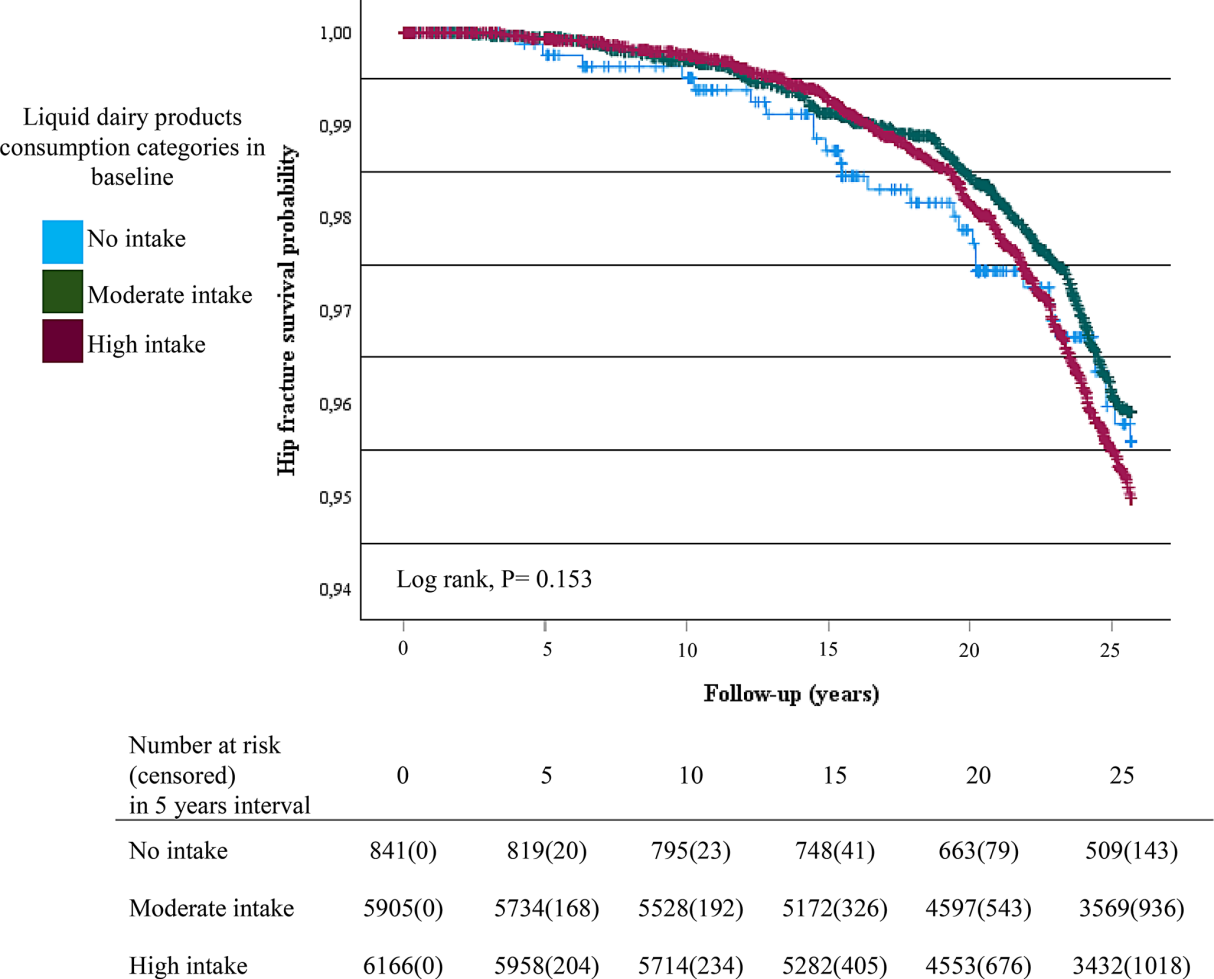
Kaplan Meier survival plot for hip fracture in categories of liquid dairy products consumption

The hip fracture survival curve did not differ significantly across categories of liquid dairy product consumption at baseline (Fig. 3). Similarly, the consumption of liquid dairy products was not significantly associated with the risk of hip fractures in time-depended analysis (Table 3). However, higher risk of hip fracture was seen in subjects who did not consume cheese at baseline compared with those who consumed moderate or high amount of cheese (data not shown). Further those women who consumed high amount of cheese had lower hazard ratio for hip fracture at 0.62 (95%CI = 0.30–0.93, P value < 0.027) compared with those who did not consume cheese in a time-dependent analysis. Further, higher consumption of cheese was also associated with a lower risk of hip fracture (β = 0.91, 95%CI = 0.83–0.99, P value = 0.043) when cheese consumption was treated as a continuous variable in a time-dependent analysis (Table 4).

## Disscussion

This long-term follow-up conducted among older Finnish women showed that higher consumption of liquid dairy products such as milk, sour milk and yogurt was associated with a lower risk of any fracture and osteoporotic fracture over 25 years. However, cheese consumption showed no association with any fracture or osteoporotic fracture risk. On the contrary, in a separate analysis using hip fracture as the outcome, liquid dairy product consumption had no association with hip fracture risk, while higher cheese consumption was associated with a lower risk of hip fracture.

### Any and osteoporotic fracture

The relationship between dairy product consumption with any and osteoporotic fracture risk has yielded mixed findings. Recent systematic reviews and meta-analyses generally indicate that high dairy intake is not significantly associated with fracture risk. For instance, a review analyzing 11 cohort and 9 case-control studies concluded that milk intake does not predict any fracture risk in either women or men across both study types [11]. Similarly, meta-analysis of 10 cohort studies revealed no association between higher consumption of dairy product and osteoporotic fracture risk [17]. Another review further supported these findings, reporting that milk or dairy product consumption does not predict osteoporosis risk [13]. The lack of evidence between dairy products consumption and risk of fracture in these reviews might be related to the insufficient data from cohort studies involving updated dairy product consumption, validated fractures, or both [12].

In contrast, large-scale cohort and randomized trial studies have found that higher dairy products consumption is linked to a reduced risk of fractures. A cohort study conducted on postmenopausal Japanese women, with a median follow-up of 15.1 years, reported that higher milk consumption was associated with a reduced risk of osteoporotic fractures; specifically, each additional cup of milk consumed per day was associated with a 4% decrease in osteoporotic fracture risk [14]. Similarly, a study involving 103,003 women over a 24-year follow-up period found that higher total dairy and milk consumption was associated with reduced risk of osteoporotic fractures, including fractures of the wrist, hip, and vertebrae [15]. A 2-year randomized trial claimed that consumption of dairy products of 3.5 servings per day was associated with a 33% reduction in any fracture risk in the intervention group compared to the control group [18].

Only one large cohort study reported a higher risk of fracture with higher consumption of dairy products. A higher risk of any fracture for women who consume more than 3 glasses of milk/day compared to women who consume one glass of milk/day has been reported by a Swedish cohort study [16]. One explanation for this finding may lie in high level of vitamin A in milk. The data for this study was collected during a period when Swedish milk was fortified by high dose of vitamin A, a nutrient linked to an increased risk of fractures at elevated levels, this might have contributed to the negative association between the consumption of milk and fracture risk [26].

There are important nutritional and physiological distinctions between cheese and liquid dairy products that support analyzing them separately. One such difference is the protein-to-calcium ratio, which can influence calcium balance and bone turnover. While milk provides a relatively balanced calcium-to-protein ratio (approximately 20:1), the same calcium intake from certain cheeses, such as cottage cheese, would result in much higher protein intake. Protein’s role in bone metabolism is complex. It promotes both calcium excretion and absorption [27]. The overall impact of cheese protein on bone health likely depends on the amount of calcium intake. Moreover, cheese contains higher sodium levels than milk or yogurt, which can further elevate urinary calcium excretion [28]. On the other hand, cheese contains several potentially beneficial components, including less D galactose and more probiotics, prebiotics, and vitamin K content compared to milk and therefore may have a more protective role against fracture than milk [26, 29, 30]. This biological rationale is supported by a growing body of literature indicating milk, yogurt, and cheese might contribute differently to fracture prevention [15, 16, 19, 20].

In our study, we did not find any association between cheese consumption and the risk of osteoporotic, or any fractures. Similarly, Kojima and his colleagues showed no evidence of an association between cheese consumption and osteoporotic fracture risk among postmenopausal women [14]. Conversely the Swedish cohort study observed that cheese consumption was linked to a reduced risk of any fractures among 61,433 women who were followed for an average of 20.1 years [16]. Yuan and her colleagues also found that higher cheese consumption was associated with a reduced osteoporotic fracture risk over 25 years of follow-up [15]. The discrepancy may be explained by the diversity in cheese types considered in different studies. Cheeses vary significantly in their compositions. For instance, the sodium content of cheese ranges widely (40–800 mg/100 g in natural cheeses, up to 1,500 mg in processed varieties) [31]. A high sodium intake leads to an increase in urinary calcium excretion and bone loss as well as increased bone remodelling [28]. Cheese fat can also vary from 20 to 35% of its dry mass [32] and those with a high content of saturated fatty acids may pose a risk to bone health [33]. Further, different types of cheese may contain varying amounts of protein [32]. As previously noted, the role of protein in bone health is complex and therefore may contribute to variation in outcomes depending on cheese type and accompanying calcium intake. Thus, it is plausible that various types of cheese consumption could have different implications for bone health. Unfortunately, the type of consumed cheese was not ascertainable in this study, hence we cannot test this hypothesis.

### Hip fracture

In this study, the association between dairy product consumption and hip fracture risk was also examined. We found that higher consumption of liquid dairy products did not affect hip fractures, whereas higher consumption of cheese was associated with lower hip fracture risk, but only in the adjusted model. Unlike our study results, higher consumption of milk among 4614 older Icelandic adults (mean age = 76 years) was associated with lower hip fracture risk in a linear way during 7 years of follow-up [21]. Similarly, according to a review study, the higher consumption of milk was associated with a 29% decrease in hip fracture risk in case-control studies [22]. An US study with 32 years of follow-up found that each additional serving of milk per day reduced the risk of hip fracture by 8% among 80,600 women and 43,306 men over the age of 50. The consumption of milk was monitored every four years during this study [19]. The lack of significant association between liquid dairy product consumption and hip fracture risk in our study could be due to an insufficient number of hip fracture events, limiting the statistical power to detect a potential relationship.

The results of our study concerning cheese consumption and hip fracture risk are consistent with the findings of two systematic reviews and meta-analyses. These reviews revealed a decreased risk of hip fractures in individuals with the highest cheese consumption compared to those with the lowest consumption in cohort studies [12, 22]. However, since there is a limited number of studies that examined the association between cheese consumption and hip fracture risk, it is difficult to draw firm conclusions.

Based on our study results, higher dairy product consumption is associated with a lower risk of osteoporotic and any fractures, but not hip fractures. This difference can be attributed to the varying effects of dairy consumption on different types of bones. The spine, which mostly contains trabecular bone, is more susceptible to vertebral compression fractures, a common feature of osteoporosis. Trabecular bone has a lower calcium content but a much higher surface-to-volume ratio than cortical bone, which increases its metabolic activity due to the higher presence of osteoblasts and osteoclasts. On the other hand, the hip consists mainly of cortical bone [34]. Therefore, calcium deficiency tends to reduce bone density more quickly in trabecular bone than in cortical bone. Thus, the protective effect of dairy products may be more pronounced in areas rich in trabecular bone, such as the spine, compared to cortical bone sites like the hip [35]. Additionally, the low prevalence of hip fractures among our study participants limited our ability to detect any association between dairy product consumption and hip fracture risk. This is to be expected, as hip fractures are more common in women aged 70–75 [7], while the mean age of our study population at baseline is 52.3 years. Therefore, further studies involving older participants are needed to investigate the effect of dairy product consumption on the risk of hip fractures.

Further, the study’s findings, particularly those shown in figures, indicate that the most significant difference in fracture risk is between the “no intake” group and the other dairy consumption categories. This difference might be attributed to a threshold effect of calcium on bone health [9]. Once this threshold is reached, even with moderate dairy intake, fracture risk decreases substantially, and any additional consumption beyond this point may either offer no further benefit or provide only minimal additional skeletal advantages.

### Strength and limitation

The main strength of this study is repeated measures of dairy products consumption and fracture incidence for 25 years. This extensive data collection allowed us to find the time-dependent association between dairy products consumption and fractures risk. Another notable strength is the substantial number of participants, that gives us enough statistical power to detect associations. However, several study limitations should be considered. First, our questionnaire captures information about the amount of dairy products consumed but does not cover the whole diet or the total energy intake. However, total energy intake is associated with BMI, which we were able to adjust. Due to limitations in the dietary data, we could not account for the fermentation status, fat content, or specific types of dairy products consumed. Further, consumption of dairy products was based on a questionnaire which are susceptible to recall error. However, the provision of recorded dietary data in 5-year intervals is likely to decrease the risk of systematic recall bias in this study. Calcium and vitamin D supplement use was categorized as a binary variable (yes/no), which limited the ability to conduct a more detailed quantitative analysis of intake levels. This may have affected the assessment of its impact on fracture risk. Another limitation of this study is the homogeneity of the study population, which may restrict the generalizability of our findings to more diverse populations.

## Conclusion

In conclusion, our results indicate that the consumption of liquid dairy products including milk, sour milk, and yogurt may decrease the overall risk of fracture, and osteoporotic fractures. Consequently, implementing public health policies aimed at promoting the recommended consumption of liquid dairy products may prevent fracture risk in the aging population, thus decreasing the associated economic burden.

**Table 1:** Characteristics of study population (*n* = 14220) presented in consumption categories of liquid dairy products and cheese

		Liquid dairy product consumption				Cheese consumption			
		No intake, 0 dl/day	Moderate intake, ≤ 4 dl/day	High intake, > 4 dl/day	*P* value*	No intake, 0 slice/ day	Moderate intake, ≤ 3 slices/day	High intake, 3 slice/day	*P* value*
Number (%) of women		842 (6.5)	5906 (45.7)	6167 (47.8)		1171 (9.2)	5745 (46.2)	5527(44.4)	
Age (years)	N	842	5906	6167	0.011	1171	5745	5527	< 0.001
	Mean (SD)	52.1 (2.8)	52.2 (2.9)	52.4 (2.9)		52.5 (2.9)	52.5 (2.9)	52.0 (2.8)	
BMI (kg/m2)	N	837	5881	6118	< 0.001	1163	5711	5498	< 0.001
	Mean (SD)	25.3 (4.0)	26.0 (4.2)	26.7 (4.5)		26.7 (5.1)	26.4 (4.4)	25.9 (4.1)	
Use of vitamin D and/or calcium supplements (yes)	N	116	386	335	< 0.001	73	355	3920	0.130
	Percentage	13.8%	6.5%	5.4%		6.2%	6.2%	7.1%	
Physical activity (hour/month)	N	446	3086	2923	0.070	506	2855	2937	0.080
	Mean (SD)	19.7 (14.2)	18.6 (13.0)	19.9 (14.0)		21.0 (15.0)	18.4 (13.4)	19.7 (13.3)	
Consumption of cheese (slice/day) or liquid dairy products (dl/day)	N	831	5671	5826	< 0.001	1168	5704	5453	0.464
	Mean (SD)	4.0 (3.3)	3.5 (2.5)	3.5 (2.6)		4.5 (3.1)	4.3 (2.6)	4.4 (2.8)	
Number (%) of women with any fracture during follow-up		289 (34.3)	1836 (31.1)	1800 (29.2)		356 (30.4)	1764 (30.7)	4.5 1696 (30.7)	
Number (%) of women with osteoporotic fracture during follow-up		172 (20.4)	1037 (17.6)	1016 (16.5)		203 (17.3)	1018 (17.7)	4.6 938 (17.0)	
Number (%) of women with hip fracture during follow-up		29 (3.4)	178 (3.0)	215 (3.5)		44 (3.8)	195 (3.4)	4.7 165 (3.0)	

_*One−way ANOVA test and respective non−parametric test (Kruskal–Wallis) was used to find means, standard deviation (SD) and P value for continuous variable_

_*Chi−square test was used to find frequency, valid percentages and P value for categorical variables_

**Table 2:** Time-dependent association between liquid dairy products and cheese consumption and any fracture

	Unadjusted				Adjusted			
	β	SE	CI	*P* value	β	SE	CI	*P* value
**Liquid dairy products (continuous)**	0.98	0.01	(0.96,0.99)	**< 0.001**	0.98	0.01	(0.96,0.99)	**0.008**
**Liquid dairy products (categorical)**								
no intake, 0	Reference	Reference	Reference		Reference	Reference	Reference	
moderate intake, ≤ 4 dl/day	0.76	0.06	(0.67,0.86)	**< 0.001**	0.77	0.08	(0.66,0.91)	**0.002**
high intake, > 4 dl/day	0.73	0.06	(0.64,0.82)	**< 0.001**	0.74	0.08	(0.62,0.87)	**< 0.001**
**Cheese (continuous)**	0.99	0.01	(0.98,1.01)	0.375	0.99	0.01	(0.97,1.01)	0.445
**Cheese (categorical)**								
no intake, 0	Reference	Reference	Reference		Reference	Reference	Reference	
moderate intake, ≤ 3 slices/day	1.00	0.06	(0.89,1.13)	0.944	1.00	0.08	(0.85,1.17)	0.996
high intake, > 3 slices/day	0.97	0.06	(0.86,1.10)	0.663	0.92	0.08	(0.79,1.08)	0.311

_The significant level of *P*<0.05 was bolded_

_β, SE (standard of error) and CI (confidence interval) were obtained from cox proportional hazard model_

_The cox proportional hazard model was adjusted for age, body mass index, intake of alcoholic beverages, use of bone affecting medications, use of vitamin D and/or calcium supplements and monthly hours of physical activity_

**Table 3:** Time-dependent association between liquid dairy products and cheese consumption and osteoporotic fracture

	Unadjusted				Adjusted			
	β	SE	CI	*P* value	β	SE	CI	*P* value
**Liquid dairy products (continuous)**	0.96	0.01	(0.95,0.98)	**< 0.001**	0.97	0.01	(0.95,0.99)	**0.011**
**Liquid dairy products (categorical)**								
no intake, 0	Reference	Reference	Reference		Reference	Reference	Reference	
moderate intake, ≤ 4 dl/day	0.74	0.09	(0.63,0.89)	**< 0.001**	0.69	0.11	(0.56,0.86)	**0.001**
high intake, > 4 dl/day	0.67	0.09	(0.57,0.80)	**< 0.001**	0.64	0.11	(0.51,0.80)	**< 0.001**
**Cheese (continuous)**	0.99	0.01	(0.97,1.01)	0.405	0.99	0.01	(0.97,1.02)	0.479
**Cheese (categorical)**								
no intake, 0	Reference	Reference	Reference		Reference	Reference	Reference	
moderate intake, ≤ 3 slices/day	0.97	0.08	(0.82,1.15)	0.738	0.92	0.11	(0.75,1.14)	0.469
high intake, > 3 slices/day	0.95	0.08	(0.81,1.12)	0.568	0.86	0.11	(0.70,1.07)	0.185

_The significant level of *P*<0.05 was bolded_

_β, SE (standard of error) and CI (confidence interval) were obtained from cox proportional hazard model_

_The cox proportional hazard model was adjusted for age, body mass index, intake of alcoholic beverages, use of bone affecting medications, use of vitamin D and/or calcium supplements and monthly hours of physical activity_

**Table 4:** Time-dependent association between liquid dairy products and cheese consumption and hip fracture

	Unadjusted				Adjusted			
	β	SE	CI	*P* value	β	SE	CI	*P* value
**Liquid dairy products (continuous)**	0.98	0.03	(0.93,1.04)	0.602	0.97	0.04	(0.90,1.05)	0.448
**Liquid dairy products (categorical)**								
no intake, 0	Reference	Reference	Reference		Reference	Reference	Reference	
moderate intake, ≤ 4 dl/day	0.86	0.32	(0.46,1.61)	0.637	0.71	0.38	(0.34,1.51)	0.380
high intake, > 4 dl/day	0.78	0.32	(0.41,1.46)	0.435	0.66	0.38	(0.31,1.40)	0.278
**Cheese (continuous)**	0.94	0.03	(0.88,1.00)	0.061	0.91	0.05	(0.83,0.997)	**0.043**
**Cheese (categorical)**								
no intake, 0	Reference	Reference	Reference		Reference	Reference	Reference	
moderate intake, ≤ 3 slices/day	0.60	0.22	(0.39,0.94)	**0.026**	0.62	0.28	(0.36,1.07)	0.089
high intake, > 3 slices/day	0.61	0.22	(0.39,0.95)	**0.028**	0.53	0.29	(0.30,0.93)	**0.027**

_The significant level of *P*<0.05 was bolded_

_β, SE (standard of error) and CI (confidence interval) were obtained from cox proportional hazard model_

_The cox proportional hazard model was adjusted for age, body mass index, intake of alcoholic beverages, use of bone affecting medications, use of vitamin D and/or calcium supplements and monthly hours of physical activity_
